# Understanding the Cognitive Immersion of Hospitality Architecture in Culture and Nature: Cultural Psychology and Neuroscience Views

**DOI:** 10.3389/fpsyg.2022.833819

**Published:** 2022-03-04

**Authors:** Haihui Xie, Qianhu Chen, Chiara Nespoli, Teresa Riso

**Affiliations:** ^1^School of Design and Architecture, Zhejiang University of Technology, Hangzhou, China; ^2^Department of Business Sciences, Alma Mater Studiorum University of Bologna, Bologna, Italy; ^3^Department of Economics and Business, University of Catania, Catania, Italy

**Keywords:** neuroscience, cultural psychology, cognitive, architecture, nature, hospitality

## Abstract

Hotel architectural design plays a critical role in the hospitality experiences of consumers, and it is important to consider that people may have different aesthetic cognitions toward the sensory properties of nature (i.e., the architecture of the hotel), such as its color and texture, as well as the landscape. While neuroaesthetics has emerged as a nascent field in hospitality research, few studies have investigated how nature reflects aesthetic experiences in the human brain. Moreover, the neuroaesthetic interpretation of architecture through the aesthetic triad is a novel interdisciplinary field. A field survey conducted at Amanfayun, a hotel in Hangzhou, China, in support of our propositions proves that sensory-motor, knowledge-meaning, and emotion valuation systems play key roles in appreciating architectural aesthetics. This study demonstrates that the evaluation of fluency, complexity, and naturalistic patterns of an architectural masterpiece is achieved through the sensory-motor systems. Our results also prove that familiarity, expectations, context, and cultural background directly affect the aesthetic knowledge of an individual and the meaning of architecture. Moreover, the interaction of sensory-motor and knowledge elements is uniformly moderated by the emotion valuation systems, resulting in a balanced appreciation of aesthetic architecture. Finally, the study reveals the central roles of culture and nature in cognitive rejuvenation.

## Introduction

More than half of humanity lives in urban areas, and this statistic will reach 70% in 2050 (Heilig, 2012). However, despite worldwide pride in urbanization, there is an unfortunate disconnect between modern architecture and natural elements, particularly those related to cultural heritage. As indicated by many scholars (Turner et al., 2004; Paraponaris and Sigal, 2015; Chatterjee and Vartanian, 2016), an unprecedented increase in urban living is associated with a lack of exposure to the cultural and natural environment, and evidence indicates that this situation coincides with an alarming global prevalence of mental health disorders (Whiteford et al., 2013).

Echoing the aforementioned mental health problems, an increasing number of studies, especially those at the intersection of neurocognition and hospitality architecture, have revealed that eco-aesthetics play a moderating role in the relationship between neurorecalibration and longevity (Turner et al., 2004; Whiteford et al., 2013). Scholars have noted that because cultural heritage and nature could bring various health benefits to human beings, hospitality architecture is experiencing a paradigm shift in which cultural and ecological landscapes are transplanted into the structural design of hospitality architecture (Rossi et al., 2020). However, although the individual imprints of culture, nature, and architectural aesthetics on the brain are well documented in the literature (Usai et al., 2020), hitherto, the joint importation of culture and nature into architecture, especially the implication on the human nervous system, has not been researched (Turner et al., 2004).

To fill this research gap, we built our theoretical basis on the theory of the aesthetic triad (i.e., sensory-motor, knowledge-meaning, and emotion valuation) using a single case study of an Aman resort in Hangzhou, China, to examine the neurological underpinning of cultural heritage and natural biodiversity reproduced in hotel architecture.

The main contribution of this study lies in using a neuroaesthetic approach to examine how the aesthetic dimension of hospitality can have a meaningful impact on human cognition. Moreover, we empirically examined the applicability of the ancient Roman aesthetic triad in a modern Chinese context. The use of the aesthetic triad scientifically established the mechanism of the cognitive process of architectural aesthetic appreciation and implied that the neuroaesthetic experience may transcend time, space, and culture.

## Theoretical Foundation: The Analytical Framework of the Aesthetic Triad

Across time and space, different civilizations have focused on different aspects of architectural design. For example, twentieth-century designs focused more on the utility aspects of fire safety, cost, and space maximization, which led to taller and sturdier buildings that mirrored the creed “form follows function” (Vitruvius et al., 1914). However, knowledge imported from neuroscience that demonstrates the undeniable impacts of aesthetics on the nervous system (Del Giudice et al., 2017; Caputo et al., 2021) has called for an architectural revolution. Consequently, beyond exploring the implications of the aesthetic qualities of architecture on cognitive functioning, behavior, and mental health (Adams, 2014; de Vasconcelos Gomes et al., 2021), architectural reform has instead become a target of neuroscience (Chatterjee and Vartanian, 2016).

While it seems difficult to capture the neuroaesthetic experience realistically and concretely, Coburn et al. (2017) proposed that the aesthetic triad established by the ancient master architect Vitruvian (Vitruvius et al., 1914) can be applied as a general neural model to embody past and future architectural aesthetic experience of a human. The three pillars of architectural design, namely, firmness (*firmitas*), usefulness (*utilitas*), and aesthetic virtue (*venustas*), demonstrate the various interplays of multiple neural networks, such as the visual, auditory, somatosensory, olfactory, and vestibular systems (Vartanian et al., 2015; Chatterjee and Vartanian, 2016). For a better understanding, the sensory-motor, knowledge-meaning, and emotion valuation systems are discussed in the following sections.

### The Sensory-Motor Neural System

To a large extent, the first contact with an architectural piece is through vision. This begins with a low-level visual identification of luminance, color, and motion, which are then accompanied by a more delicate process involving the parahippocampal, occipital, and retrosplenial cortex areas (Vartanian et al., 2015). While the parahippocampal cortex captures the scene in the environment, the occipital region retains fixed features in the spatial environment, and the retrosplenial cortex remembers the elements and helps orient the individual. However, architectural works also have non-visual effects, such as odor, sound, and temperature, which directly involve the emotion of the individual (Barbara and Perliss, 2006).

Another important consideration is the neural motor responses that coordinate parts of the neural system to respond to different scenes (Papa et al., 2018a,b). The motor system coordinates the nucleus accumbent and the anterior insula, which dictates whether an architectural piece should be approached or avoided (Vartanian et al., 2015). In other words, appreciation of people of architectural aesthetics is intrinsic to the impact of previous experiences constructed through culture and nature on cognitive mechanisms and functions. The chain of functioning is closely linked to the meaning-knowledge systems built on personal experiences, culture, and education (Lugmayr et al., 2017).

### The Knowledge-Meaning System

Appreciation of a person of an architectural object is directly related to their memories and past experiences. In particular, past experiences in a cultural setting directly modulate present interactions and interpretations of a person of architectural aesthetics. Neuroaesthetic findings show that cultural experience engages a cognitive map involving the cells of the hippocampus (McNaughton et al., 2006). Grid cells of the hippocampus function as encoded memories of both events and places, thereby building a level of familiarity to help with identification and navigation. Cultural background also has a direct impact on the expectations of architectural quality and value of a person (Duan et al., 2021). The aesthetic quality or value of an architectural object can increase or decrease the level of comfort of a person through simple perception (McNaughton et al., 2006; Sharma et al., 2021).

An architectural design is likelier to embody an emotional reaction if it is related to the cultural background or past experience of a person than random computer-generated images (Kirk et al., 2009). Studies show that culture-related emotions are known to increase activities in the prefrontal, orbitofrontal, and entorhinal cortices, while computer-generated designs have minimal to no reaction in those responses of the brain (Caputo et al., 2018; Chin et al., 2021b).

Consequently, the response of the sensory-motor and knowledge-evaluation systems needs the regulating principle of the emotion valuation system, which functions to balance feelings and emotions generated by architectural aesthetics. According to the fundamental judgment capacity of the brain, emotions are regulated, controlled, and balanced in a manner to avoid excesses, either negative or positive.

### The Emotion Valuation System

Studies have overwhelmingly demonstrated how exposure to nature improves the functioning of the emotion evaluation system (Adams, 2014). As a pivotal system that mediates the operation of other systems in the architectural aesthetic experience, the emotion valuation system relies on frequent exposure to nature for constant cleansing and rejuvenation.

Several studies have shown that interactions with nature improve the emotion valuation ability of the brain (Cimprich, 1992, 1993). A study that used the same design parameter in two different environments (one enclosed and the other open) revealed that enclosure produced a higher degree of fear and stress hormones (Paraponaris and Sigal, 2015) because the emotion-regulating limbic structures of the neuroendocrine and autonomic nervous systems were not refreshed (Cimprich, 1992). Other studies have also revealed that positive emotional responses occur more rapidly and automatically in a natural environment than in an enclosed environment (Cimprich, 1993; García-Villaverde et al., 2018; Chin et al., 2020).

**FIGURE 1:**
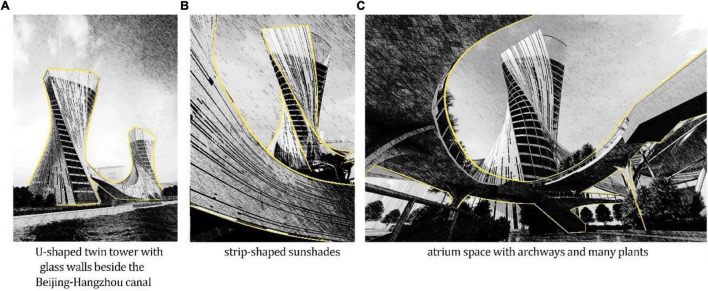
The architectural design of a commercial complex.

Given these arguments, it is obvious that aesthetic judgments of individuals of architecture largely vary with brain activities in the frontopolar cortex, superior frontal gyrus (the pFC region), and memory retrieval regions (Vartanian et al., 2013). Thus, we proposed that such aesthetic judgment is involved with the conscious and analytical reasoning of individuals, which is shaped by their cultural backgrounds. To more explicitly address our arguments, an architectural design plan made by our first author (Haihui Xie) delineates how an architectural design carrying unique cultural meaning immerses itself in its natural surroundings (Figure 1).

## Development of Three Propositions

### A Brief Description of the Architectural Design Used

As shown in Figure 1, the architecture design draft is a *commercial complex that includes office buildings, malls, and the like beside the Beijing–Hangzhou Grand canal*.

This design offers precise portraits of architectural aesthetics that are a combination of culture and nature. From an aesthetic perspective, not only does the vertical alignment of the towers blend with the horizontal flow of the river at the bottom but also the transparent and reflexive properties of the glass and water create a peculiar emersion that can be better appreciated through a cognitive interpretation.

Based on this draft, three propositions about the aesthetic judgment criteria for sensory-motor, knowledge experience, and emotion valuation, respectively, are developed and discussed in the following sections.

### Proposition 1: Sensory-Motor Systems

Fluency includes spatial and visual aspects. While the spatial aspect is reflected in the vertical alignment of the building beside a flowing river, the visual concept can be shown as translucent or transparent material in architecture (Coburn et al., 2019). As shown in Figure 1A, there are two tall towers with a winding superstructure made of glass built right at the side of a famous river with remarkable historical and cultural backgrounds. Apart from its alignment with nature, the fluency in this masterpiece is further demonstrated by the building and the river reflecting off each other. When the reflection of the building can be seen in the river, the flow of the river is simultaneously reflected in the glass walls of the towers.

Berlyne (1971) stated that the complexity of a pattern increases with the increasing number of independently selected elements it contains. A building of moderate complexity should have harmonious unity with several design levels or methods, meaning that it should have a logical hierarchy. In this plan, as shown in Figure 1A, the building was designed to have a torsional body that is bent into U-shaped twin towers with repetitive, grouped, and strip-shaped sunshades, flowing along with the entire building. Therefore, the facade of the building would have three levels: macroscopic, middlescopic, and microscopic.

The sensory qualities of natural environments have been found to improve mood, attention, and cognitive functioning (Kaplan, 1995; Berman et al., 2008; Bratman et al., 2012, 2015), among other salubrious effects. Kellert (2005) defined organic design as “building shapes and forms that directly, indirectly and symbolically elicit a human affinity for natural features and processes” (p. 128), which was proposed to confer psychological benefits similar to interacting with nature itself (Alexander, 2002; Kellert, 2005; Joye, 2007; Salingaros, 2007; Coburn et al., 2019). In this plan, there would be plentiful trees and shrubbery planted in the atrium space. With the curvilinear boundary of the building, people would be willing to visit it, as shown in Figure 1C.

Based on the earlier arguments, we proposed the following:

**Proposition 1** for the sensory-motor systems: Motor responses of people to architecture can be visually evaluated by fluency, complexity, and naturalistic patterns.

### Proposition 2: Knowledge-Meaning Systems

Zajonc (1968) hypothesized that individuals should fear the unknown. On repeated exposure, a linear increase in positive affect is produced by greater familiarity and reduced uncertainty (called “stimulus habituation”) (Montoya et al., 2017). Therefore, in architecture, familiarity can be shown as an easy route and a familiar scene space. In this plan, the flow of people in the commercial complex was designed to be circular, which fits the behavior of people and reduces their uncertainty. The building was also planned to show spaces that reflected traditional buildings in Hangzhou, such as archways, as shown in Figure 1C.

Expectations about control influence thermal comfort. Occupants who can control environmental parameters affecting building temperatures, such as operable windows, fans, and thermostats, tolerate a wider range of indoor temperatures than inhabitants with restricted control over their indoor climate (Nicol and Humphreys, 2002; Coburn et al., 2017). As shown in Figure 1B, the sunshades outside the glass curtain wall could be adjusted to control the indoor temperature, given that the intensity and direction of the sun would change.

Art, including architectural aesthetics, provides numerous solutions to the problem of meaning assignment. Thus, in every case, the internal evidence measured at the evaluative level determines the aesthetic experience (Leder et al., 2004), and cultural meaning affects the aesthetic experience (Chin et al., 2021a). In our design, for instance, the cultural connotation is reflected in the flowing sunshades in the plan, which, when reflecting the sunshine, show the canal glinting in the sun, as shown in Figure 1B.

Based on the earlier discussion, we proposed the following:

**Proposition 2** for the knowledge-meaning systems: Aesthetic experience of people of space can be affected by familiarity, expectations, context, and cultural meaning.

### Proposition 3: Emotion Valuation Systems

From the study by Coburn et al. (2017) about emotion valuation systems, the architectural experience, which is mediated by the reward circuitry of the brain, can be divided into two dimensions: Evaluative and emotional responses to architecture. The former is influenced by input from knowledge-meaning systems, and the latter might be influenced by the automatic, unconscious sensory-motor process.

Consequently, the design in this case combined all elements of sensory-motor and knowledge-meaning, resulting in an emotional landmark immersed in culture and nature. Eventually, the reactions of the sensory-motor and knowledge evaluation systems necessitate interaction with emotion valuation systems, which function as a regulator to balance feelings and emotions generated by architecture (Kirk et al., 2009; Wang et al., 2020). This is the fundamental judgment capacity of the brain, where the emotion projected by aesthetics is to regulate, control, and balance in a manner that avoids excesses, either negative or positive.

**Proposition 3** for the emotion-valuation system: The interactions of the sensory-motor and the knowledge-meaning systems mentioned in Propositions 1 and 2 can determine the neural underpinnings of complex emotional valuation.

## Methodology

### Sample Selection: The Luxury Amanfayun Hotel in Hangzhou, China

Scholars have noted that a single case study is appropriate when the selected case is unique and extreme. Thus, in a single case study, sample selection seems to be the most critical procedure because the research sample must be specific and representative of all cases. Considering the exploratory and distinctive nature of our research, we adopted a single-case research design.

After reviewing well-known multinational hospitality companies in China, we carefully selected Amanfayun, which is located in Hangzhou, China, and is owned by the Aman group (a renowned Indonesian hospitality company) as the case hotel. The choice of an Aman hotel was due to the particular representativeness of the modest luxury of the group in the global hotel industry.

The Aman group built its first luxury hotel in Phuket in 1988 and has expanded its resort business to other countries in Asia and to America, Africa, and Europe over the last three decades. Aman resorts are known for buildings and landscapes that are designed to merge with the local cultural and natural contexts. For example, the white colonnades and pediments in the hotel of Aman in Greece are reminiscent of the Parthenon, while the luxury Rococo decoration in the Venice hotel of Aman shows the signature style of the city to guests. Apart from the diversity of hotels of Aman in different countries, the hotels of the group in China show differences in architecture from district to district. For example, the Aman Summer Palace, which is beside an ancient royal garden in Beijing, has quadrangle dwellings, Dougong brackets, and other features of the ancient architecture of Northern China, while Amanyangyun, which is located in suburban Shanghai, shows more characteristics of the ancient civil architecture of Southern China, such as Chinese gabled rooves and through-jointed frames.

It is relatively difficult to travel across cities amid the pandemic. Given that our research team is based in Hangzhou, China, we selected Amanfayun, which is located on a mountain in Hangzhou, as our research sample for the field survey. This hotel is built in the vicinity of the famous Lingyin Buddhist Temple, which was built in the third century BC and has been officially recognized as one of the key cultural relics of China requiring protection.

### Data Collection

The main form of data collection was a field survey. The goal was to collect information at the local level by conducting primary surveys. We organized a team of six researchers who visited and experienced the surrounding areas while they stayed at Amanfayun for 3 days. The researchers walked around to locate and observe special points of the architecture and landscape at Amanfayun relating to the aforementioned aesthetic triad.

To enhance methodological rigor, we also conducted in-depth interviews with selected hotel guests with different cultural backgrounds. During the interviews, which were considered a pilot study prior to the formal survey, we spoke freely with the interviewees and let them describe their aesthetic appreciation of the hotel space and architecture. Their feedback was consistent with our arguments and research framework, confirming that our propositions were logical. The interviewees are listed in Table 1.

**TABLE 1:** Overview of the interview data.

No.	Name	Job	Gender	Age	Domicile
1	Lily	College undergraduate	F	21	Hangzhou, Zhejiang, China
2	Huang	Post-doctor	M	30	Hangzhou, Zhejiang, China
3	Vivian	Tourism blogger	F	24	Hong Kong, China

### Results of the Interview

The first interviewee was an undergraduate student majoring in architecture at a university in Hangzhou. She had come to the hotel environment to research hospitality buildings and to study cases for an upcoming design workshop. The moment she stepped into Amanfayun, she felt that it was a tranquil and healing environment. She thought that the architecture looked similar to civilian buildings in mountain areas in Zhejiang. While she found it comfortable to walk on the main road of Amanfayun, she was not sure whether people who chose to stay in the suites near the main road would not be disturbed by the traffic. After learning that the cost of one of the cheapest suites at Amanfayun for one night would be more than 5,000 RMB, she stated that she would travel here for that cost.

The second interviewee, a Ph.D. candidate, had been passing by on his way to the Lingyin Temple and thought that the hotel would be a good place to spend the holiday. He found it comfortable and tranquil in the natural environment away from the city and thought that the temples beside the hotel allowed tourists to relate to Buddhist culture. When observing the views, he felt connected to lands mentioned in ancient Chinese poetry. However, he thought the price was too high, suggesting that a reasonable price would be 500–600 RMB. Moreover, he thought the living experience might not be optimal because of the many tourists visiting the hotel area.

The third interviewee was a tourism blogger from Hong Kong who ordered a suite at Amanfayun. She thought it was worthwhile to stay in the hotel because of its close location to Lingyin Temple. Additionally, while some people might think the suite buildings were too plain for the price, she said that the buildings reflected 100 years of history, suited the natural environment, and felt cozy and quaint. The beautiful environment made her feel like a hermit on the mountain. When asked about the influence of tourists traveling to Lingyin Temple on the main road of the hotel, she said it was acceptable because few tourists were passing by and they could not enter the main living area of the hotel guests.

## Findings of the Field Survey

**FIGURE 2:**
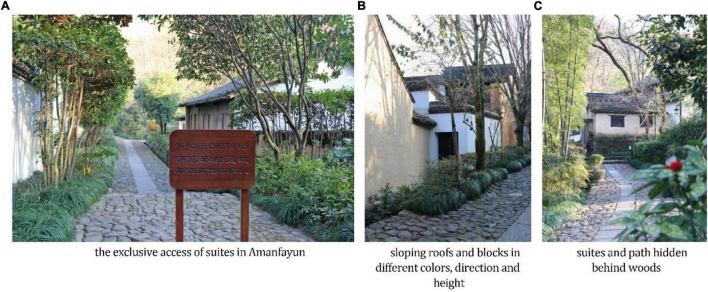
Photos of Amanfayun verifying Proposition 1.

In terms of fluency, the suites at Amanfayun are connected with the main road of the hotel and insulated by a notice board, fence, and staff (Figure 2A), notifying people not to pass but not occluding the view of the hotel.

As for complexity, the sloping rooves of blocks in different directions, heights, and colors (Figure 2B) show the design hierarchy. All the buildings of Amanfayun are unified in texture and style, giving the hotel a harmonious appearance.

Regarding naturalistic patterns, the landscape includes a view of Mount Feilai. The suites and paths are hidden in woods, as shown in Figure 2C, with a stream beside them. There are also natural design elements that have a traditional Chinese aesthetic, such as bonsai and rocky stones.

**FIGURE 3:**
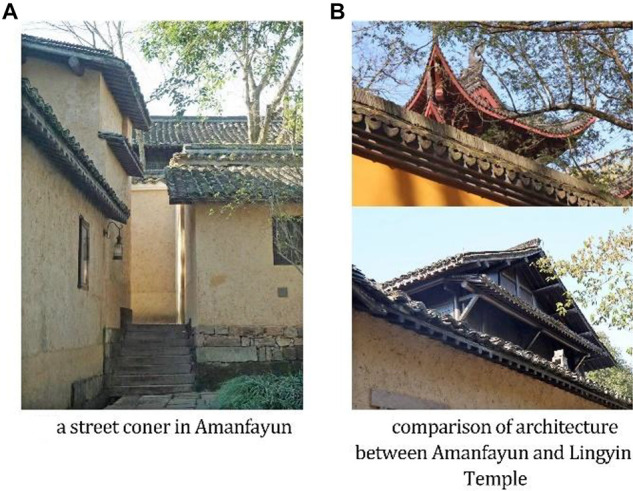
Photos of Amanfayun verifying Proposition 2.

For familiarity, we mentioned the easy route and familiar scene above. In Figure 2, the structure of the hotel is clear. Each suite, along with its yard and exclusive access, is connected by the main road that starts at Zhejiang Buddhist College and ends at Lingyin Temple. Figure 3A shows a corner of Amanfayun that is similar to a typical street scene of traditional dwellings in Zhejiang.

To satisfy the expectations of guests about controlling the environment, the indoor suites at Amanfayun were designed to combine elegance and comfort^1^. There are modified curtains, lamps, and other electric appliances with a retro look for guests to adjust to the indoor environment. Discordant modern electric appliances, such as air conditioners, are in inconspicuous positions.

As for context and cultural meaning, Amanfayun is close to Lingyin Temple, a famous Buddhist temple with almost 1,700 years of history, and Zhejiang Buddhist College. Therefore, the architecture and landscape of Amanfayun echo the buildings in the temple and the college, as shown in Figure 3B. The environment at Amanfayun reflects the serenity of Buddhism in a common way. The outer wall of the buildings is yellow (the same color as the Lingyin Temple), but it is made of earth to achieve a cozy feel.

## Discussion and Conclusion

Even though aesthetics plays an important role in modern architecture, the roles of culture and nature have been neglected. The literature shows a direct correlation between architectural designs and psychological challenges with occupants (Chatterjee and Vartanian, 2016). To demonstrate the role of culture and nature in architectural aesthetics and the implications for the nervous system, we conducted a field study to demonstrate three propositions built on the famous aesthetics triad.

Using the Amanfayun hotel in Hangzhou as a case study, we established a neuroaesthetic appreciation of architectural designs based on the sensory-motor, knowledge-meaning, and emotion valuation systems, as described by the aesthetic triad. This study demonstrates that the evaluation of fluency, complexity, and naturalistic patterns of an architectural masterpiece can be achieved through the sensory-motor systems. Our results also prove that familiarity, expectations, context, and cultural background directly affect the aesthetic knowledge of an individual and the meaning of architectural work. Moreover, the interaction of sensory-motor and knowledge elements is uniformly moderated by the emotion valuation system, resulting in a balanced appreciation of aesthetic architecture.

The conceptual analysis used in this study contributes to the neuroaesthetic and architecture literature by conducting a single case research study using the Aman resort groups to investigate the fusion of culture, nature, and architecture and the implications of cultural and ecological submersion on the human nervous system. To deepen our study, we used the aesthetic triad concept to establish the role of neuroaesthetics in interpreting architecture.

It seems that the neuroaesthetic approach to architectural appreciation is founded on the aesthetic triad systems of the brain. Moreover, the submersion of architecture into culture and nature reveals a superior paradigm of cognitive interaction and neural wellbeing. After establishing the central role of the aesthetic triad in evaluating aesthetic architecture, this discussion focuses on the immersion of culture and nature into architecture and its association with the degree of life satisfaction and wellbeing, better performance on tasks, impulse inhibition, selective attention, and concentration. More than the proven impact of the aesthetic triad, a wide range of health benefits from cultural preferences and nature exposure has been adequately proven in the literature (Ulrich et al., 1991). The impact of exposure to nature varies according to the types of contact (photographs, everyday window views, and contact with the natural environment) and the duration of the exposure.

To deepen this discussion, the research elaborates on the restorative value of being embedded in nature and its refreshing impact on the emotion valuation system. Two major theories can fundamentally evaluate the restorative influence of nature: Stress reduction theory (SRT) and attention restoration theory (ART) (Ulrich et al., 1991). According to SRT, there is a wide gap between exposure to natural environments and artificial artifacts, where the restorative advantage of natural environments can be attributed to the impact of evolution on the human brain (Ulrich, 1981). The theory explains that natural scenes are responsible for activating the parasympathetic nervous system, which automatically reduces stress due to its inherent relationship with the natural world. A set of testable hypotheses provided by Ulrich’s theory has been investigated *via* the physiological responses of individuals exposed to different natural environments to evaluate the impact of nature on the autonomic nervous system. Further findings to support SRT revealed that walking through a forest landscape reduces cortisol levels, mental distress, and stress levels and increases psychological wellbeing (Ulrich, 1981).

In contrast, ART analyzes the kinds of environments that improve directed-attention abilities. Nature is filled with stimuli that modestly draw the attention of an individual in a bottom-up direction and allow top-down attention to have a timed lap to replenish. This is contrary to urban environments, which are filled with constant, dramatic attention-holder stimuli that do not allow for recovery time, such as a car or people suddenly approaching. According to ART, nature attracts the attention of people in many fascinating ways, resulting in the rejuvenation of directed attention and memory. In support of this, Cimprich (1992) showed that students who had their windows open to a natural environment performed better on high-concentration tasks than those with no view of nature. Similarly, walking through a natural green space is beneficial to verbal working memory, cognitive control, and concentration. The results cited are a fraction of the findings demonstrating the health benefits of exposure to nature, especially in terms of cognitive performance on memory and direction tasks (Ulrich, 1981).

## Data Availability Statement

The original contributions presented in the study are included in the article/supplementary material, further inquiries can be directed to the corresponding author/s.

## Author Contributions

HX designed and conducted the research, collected the data, and wrote the main part of the manuscript. QC guided throughout the entire research process. CN and TR wrote the remaining parts and offered modification suggestions. All authors listed have made a substantial, direct, and intellectual contribution to the work, and approved it for publication.

## Conflict of Interest

The authors declare that the research was conducted in the absence of any commercial or financial relationships that could be construed as a potential conflict of interest.

## Publisher’s Note

All claims expressed in this article are solely those of the authors and do not necessarily represent those of their affiliated organizations, or those of the publisher, the editors and the reviewers. Any product that may be evaluated in this article, or claim that may be made by its manufacturer, is not guaranteed or endorsed by the publisher.

## Footnotes

1
https://720yun.com/t/a6vkOh27rih?scene_id=57462037
